# The Elapsed Time between Dinner and the Midpoint of Sleep Is Associated with Adiposity in Young Women

**DOI:** 10.3390/nu12020410

**Published:** 2020-02-05

**Authors:** María Fernanda Zerón-Rugerio, Giovana Longo-Silva, Álvaro Hernáez, Ana Eugenia Ortega-Regules, Trinitat Cambras, Maria Izquierdo-Pulido

**Affiliations:** 1 Department of Nutrition, Food Science and Gastronomy, School of Pharmacy and Food Science, University of Barcelona, 08921 Barcelona, Spain; fernanda.zeron@ub.edu (M.F.Z.-R.); giovana_longo@yahoo.com.br (G.L.-S.); 2 INSA-UB, Nutrition and Food Safety Research Institute, University of Barcelona, 08921 Barcelona, Spain; 3 Faculty of Nutrition, Federal University of Alagoas, Maceio 57072-900, Brazil; 4 August Pi i Sunyer Biomedical Research Institute (IDIBAPS), 08036 Barcelona, Spain; alvaro.hernaez1@gmail.com; 5 CIBER Physiopathology of Obesity and Nutrition (CIBEROBN), Instituto de Salud Carlos III, 28029 Madrid, Spain; 6 Department of Health Sciences, Universidad de las Americas Puebla, Cholula 72810, Mexico; ana.ortega@udlap.mx; 7 Department of Biochemistry and Physiology, School of Pharmacy and Food Science, University of Barcelona, 08028 Barcelona, Spain; cambras@ub.edu

**Keywords:** sleep timing, midpoint of sleep, meal timing, body mass index, adiposity

## Abstract

Meal timing relative to sleep/wake schedules is relevant in the search for obesity risk factors. However, clock time does not accurately characterize the timing of food intake in the context of internal circadian timing. Therefore, we studied elapsed between dinner and the midpoint of sleep (TDM) as a practical approach to evaluate meal timing relative to internal timing, and its implications on obesity. To do so, adiposity, sleep, diet, physical activity, and TDM were measured in 133 women. The participants were grouped into four categories according to their sleep timing behavior (early-bed/early-rise; early-bed/late-rise; late-bed/early-rise; late-bed/late-rise). Differences among the categories were tested using ANOVA, while restricted cubic splines were calculated to study the association between TDM and adiposity. Our results show that, although participants had dinner at about the same time, those that had the shortest TDM (early-bed/early-rise group) were found to have significantly higher BMI and waist circumference values (2.3 kg/m^2^ and 5.2 cm) than the other groups. In addition, a TDM of 6 h was associated with the lowest values of adiposity. The TDM could be a practical approach to personalizing meal timing based on individual sleep/wake schedules. Thus, according to our findings, dining 6 h before the midpoint of sleep is an important finding and could be vital for future nutritional recommendations and for obesity prevention and treatment.

## 1. Introduction

The coordination and timing of specific behaviors (e.g., sleeping and fasting) with the external light/dark cycle is essential for heath [1,2]. Therefore, the circadian system has evolved to allow adaptation of organisms to the external changing world. The temporal regulation of metabolism is coordinated through several oscillating networks, including sleep/wake homeostasis, feeding and fasting rhythms, and the action of circadian clocks [2]. However, since the invention of electrical lighting, humans have been free to select their own light/dark cycles and extend wakefulness activities far into the night, which leads to a disruption between behavior and internal circadian time, known as circadian misalignment [3].

Circadian misalignment is associated with adverse health outcomes, including impaired glucose metabolism and obesity [3]. The most extreme example of circadian misalignment is seen in people that do shiftwork. In addition, adolescents and college-aged individuals push activities to a later clock time, and thus, present a greater risk of a mild kind of circadian misalignment, denominated *social jet lag* [3]. The latter is a condition characterized by discrepancy in sleep/wake schedules on weekends versus weekdays [4]. Importantly, social jet lag is considered a potential risk factor for obesity and unhealthy dietary habits among young adults, as was recently shown by our research group [5].

College is also a stage of life frequently linked to unstructured food habits, possible excess of alcohol intake, decreased physical activity, and erratic sleep/wake patterns [5,6]. Sleep/wake schedules, referred to as sleep timing behavior, are influenced by the individual circadian preference, or chronotype, and by external factors, including the need to rise early to attend school [7]. Besides circadian misalignment, erratic sleep timing behavior may result in short sleep duration, which leads to extended hours of wakefulness, and additional opportunities to eat at inappropriate times (e.g., late night or early morning) [8]. Moreover, evidence from cross-sectional studies has shown that, among children and adolescents, late bed and late wakeup timings are associated with a higher BMI, lower physical activity, and poor diet quality [7,9,10]. Nevertheless, the association of sleep timing behavior during the week with BMI, dietary intake (energy and nutrients), and physical activity remains unexplored among college students.

Besides, emerging evidence suggests that the timing of food intake relative to sleep timing behavior could also have a negative impact on BMI [3,11]. For example, cross-sectional studies have reported that eating dinner less than two hours before bedtime is significantly associated with hyperglycemia, and higher odds of being overweight or obese [12,13]. In consonance, Garaulet and colleagues [14] showed that late dining resulted in impaired glucose tolerance, mainly due to concurrence between the postprandial period and endogenous melatonin concentrations. However, a limitation of these studies is the use of clock time to characterize the timing of food intake, which fails to accurately characterize meal timing in the context of internal circadian timing [3,11].

To address this limitation, McHill et al. [3] studied the association between the timing of food intake and dim light melatonin onset (DLMO), a marker of internal circadian time. The authors demonstrated that eating closer to, or after, DLMO was significantly associated with higher body fat, independent of dietary intake and the level of physical activity [3]. This approach, however, requires repeated blood or saliva collection to evaluate DLMO, and participants need to stay in dim light conditions for many hours, which is not practical for most epidemiological studies [11]. Therefore, Xiao et al. [11] proposed defining meal timing taking into consideration the timing of sleep/wake cycles as a proxy for internal circadian timing. Accordingly, the authors divided the waking period into four time windows, and showed that a lower dietary intake after wakeup, and higher food intake closer to bedtime was associated with higher BMI. However, the authors pointed out that this relationship between sleep/wake timing and meal timing differed according to chronotype.

Taking into account the aforementioned, we herewith measured the elapsed time between dinner and the midpoint of sleep (TDM) as a practical approach to examine the timing of food intake relative to internal circadian timing, and their implications for obesity. It is important to note that the midpoint of sleep has the highest correlation with DLMO, and is also considered to be a marker of the chronotype [4,15,16]. Our aim was to study whether TDM and sleep timing behavior were associated with anthropometric markers of adiposity. Additionally, dietary intake and physical activity were studied.

## 2. Materials and Methods

### 2.1. Study Population

Women (18–25 years) were recruited from undergraduate students at the Universidad de las Americas Puebla (Mexico) for a cross-sectional study during the school year, between August and October 2018. Exclusion criteria consisted of the inability to provide information required for the development of the study, or being previously diagnosed with Type 2 diabetes, hypertension, and/or cardiovascular disease. Based on these criteria, a total of 143 individuals were eligible, who provided written informed consent before joining this study. We further excluded subjects with missing information, resulting in a final analytical cohort of 133 subjects. All study procedures were conducted according to the Declaration of Helsinki, and were approved by the Ethics Committee at the University of Barcelona (IRB00003099), and by the Ethics and Research Committee, Department of Health Sciences, Universidad de las Americas Puebla.

### 2.2. Anthropometric Parameters

Weight and body composition were determined with a medical body composition analyzer (mBCA 514, Seca, Hamburg, Germany), with the subjects wearing light clothes and without shoes, to the nearest 0.1 kg. Height was determined using a fixed wall stadiometer (Seca 217, Seca) to the nearest 0.1 cm. BMI was calculated as weight (kg) divided by squared height (m) [17]. Waist and hip circumferences were measured using a flexible steel anthropometric tape (Lufkin, W606PM), to the nearest 0.1 cm and calibrated in centimeters. Waist circumference was measured midway between the lower rib margin and the iliac crest with the subject standing and wearing only underwear, at the end of gentle expiration [18]. Hip circumference was measured at the level of the greater trochanter, at the widest portion of the buttocks [18].

### 2.3. Sleep and Circadian Related Variables

Participants completed a 6-day sleep diary on consecutive days (including 3 weekdays and 2 weekend days) in which they recorded bedtime and wakeup timing. From these data, we calculated the following variables:

I. Sleep duration (in hours) was calculated for each day as the difference between bedtime and wakeup timing. A total weekly sleep duration was calculated as follows: [(5 × sleep duration on weekdays) + (2 × sleep duration on weekends)]/7 [19].
II. The midpoint of sleep (local time), defined as the middle time point between bedtime and wakeup timing [15]. The average time of the midpoint of sleep during the week was calculated as follows: [(5 × midpoint of sleep on weekdays) + (2 × midpoint of sleep on weekends)]/7 [19].
III. Sleep timing behavior was categorized using the median splits of the time in which each volunteer went to bed and woke up during the week [7]. First, bedtime was classified as follows: “Early-bedtime” (<23:48 h) and “Late-bedtime” (≥23:48 h). Second, for each bedtime group, we used median splits of wakeup timing. Early-bedtime subjects were divided into “Early-rise” (wakeup time <7:12 h) and “Late-rise” (wakeup time ≥ 7:12 h). Subsequently, “Late-bedtime” subjects were divided into “Early-rise” (wakeup time <7:52 h) and “Late-rise” (wakeup time ≥ 7:52 h). Accordingly, four sleep timing behavior categories were defined: early-bedtime/early-rise (EE), early-bedtime/late-rise (EL), late-bedtime/early-rise (LE), and late-bedtime/late-rise (LL).
IV. Sleep quality was measured using the Spanish version of the Pittsburgh Sleep Quality Index (PSQI) [20]. Scores range from 0 to 21, where the higher the score, the worse the sleep quality.
V. Social jet lag was measured in hours, by subtracting each participant’s midpoint of sleep on weekdays, from the midpoint of sleep on weekends [21]. All analyses were conducted using the absolute value of social jet lag [5,21].

### 2.4. Meal Timing

Meal timing was assessed with 6-day food logs, which were filled on consecutive days and included the weekend. This allowed us to evaluate the time in which each food or beverage was consumed. Meals were classified as breakfast, lunch, or dinner, based upon the designation that each participant indicated.

The average for the timing of the main meals (breakfast, lunch, or dinner) during the week was calculated as a weighted mean as follows: [(5 *× meal timing on weekdays*) + (2 *× meal timing on weekends*)]/7.

### 2.5. Time Elapsed between Dinner and the Midpoint of Sleep TDM

The TDM was defined as the timing of dinner relative to internal circadian timing. This parameter was calculated, for each individual, considering the average values of the midpoint of sleep and dinner timing, as follows: [(*midpoint of sleep +* 24) *− timing of dinner*].

### 2.6. Dietary Intake

Dietary intake was assessed with 6-day food logs, which were filled on consecutive days and included the weekend. Participants were taught by a nutritionist to record the type of food with brand name if possible, portion size, location of the meal (i.e., home, or restaurant). All meals were coded in duplicate by two nutritionists to determine food item and portion size, and any discrepancies were solved to minimize error before calculating nutrient intake. Daily intakes of energy and nutrients were calculated using PCN Pro 1.0, on the basis of Mexican food composition tables [22,23].

Additionally, diet quality was assessed using the Quality Index Food Consumption Pattern [24], a validated food-based scoring system for Mexican population. This tool evaluated daily, weekly, and occasional consumption of vegetables, fruits, grains and derivates, dairy products, meats, legumes, cold-processed meats, sweets, and beverages. Briefly, the questionnaire included questions like: “Do you consume vegetables/fruits/grains and dairy products on a daily basis?”, “Do you consume legumes/meats on a weekly basis?”, and “Do you consume occasionally processed meats/sweets/soft-drinks?” Each item was punctuated separately, oscillating from 0 to 10, with 0 being the lowest score associated with unhealthy dietary habits (e.g., never consuming fruits or daily consumption of sweets or soft-drinks), and 10 being the highest score associated with healthy dietary patterns (e.g., daily consumption of fruits or never consuming sweets or soft drinks). Subsequently, the scores were summed up, ranging from 0 to 93, where the higher the score, the better the diet quality.

### 2.7. Physical Activity

The level of physical activity was measured with the short version of the International Physical Activity Questionnaire (IPAQ) in Metabolic Equivalents of Task (MET) per week [25]. This questionnaire has been validated and has demonstrated a good correlation with accelerometer data [25].

### 2.8. Sample Size Calculation

In sleep timing behavior analysis, a sample size of 33 participants per group allowed ≥80% power to detect a significant difference of ≥2.8 kg/m^2^ in BMI between groups. We considered a 2-sided type I error of 0.05, a loss rate of 1%, and a standard deviation of BMI values similar to the one present in our population (SD = 4). In relation to general association analyses, assuming the same loss rate and type I and II errors, a sample size of 103 subjects provided sufficient statistical power to determine that Pearson’s correlation analyses ≥0.275 were significantly different from zero (sample size was increased by 30%, up to 133 individuals, to allow adjustments for different covariates).

### 2.9. Statistical Analyses

Normality was confirmed in all variables by histograms and Q-Q plots. Normally distributed variables were described by means and standard deviations and non-normally distributed variables by medians and interquartile range. We compared the values of anthropometric, sleep and circadian variable, TDM, dietary intake, and physical activity across sleep timing behavior categories by ANOVA tests for normally distributed data or Kruskall-Wallis tests for non-normally distributed variables, followed by Bonferroni post-hoc comparisons. Pearson’s correlations tests were used to determine whether there was a significant linear trend in the values of anthropometric and lifestyle variables in increasing wakeup timing across sleep timing behavior categories (reported as p-trend values). Differences in anthropometric markers between extreme sleep timing behavior categories (LL vs. EE) and +1 h increments in TDM values were tested using multivariate linear regression models. Restricted cubic splines were used to study the shape of the association between the TDM and anthropometric markers. We set the reference cut-point at the minimum value of the time between dinner and midpoint of sleep reported in our population (3.45 h). Splines were fitted and plotted using the “glm” package in R Software [26]. Multivariate linear regressions and restricted cubic splines were adjusted for age, diet quality, total energy intake, and physical activity level. Significance testing was considered when *p* < 0.05. All analyses were performed with R Software, version 3.4.1 (*R Foundation for Statistical Computing, Vienna, Austria*).

## 3. Results

The general characteristics of the studied population are shown in supplementary Table S1. Overall, 25.5% of the individuals were overweight or obese, 85.8% of the individuals showed an average diet quality, and 55.2% reported moderate physical activity. Notably, 70.7% of the individuals included in our study did not reach the recommended 7 h of sleep per night [27]. Details about average sleep and meal timing are provided in Table S1.

Sleep- and circadian-related variables are summarized in Table 1, grouped by sleep timing behavior categories. As expected, individuals in the EE group had the earliest wakeup timing and midpoint of sleep, whereas individuals in the LL group showed considerably later timing of wakeup timing and midpoint of sleep (*p* < 0.001). In addition, a significant trend towards later wakeup time, bedtime, and midpoint of sleep was observed across the four categories (*p* < 0.001). We also observed a significant trend towards longer sleep as the wakeup timing was delayed (*p* < 0.001): those groups that woke up earlier were those with the shortest sleep duration. Of note, sleep quality and social jet lag were similar among groups.

Breakfast was the meal best associated with sleep timing behavior, while dinner timing was not modified by sleep timing behavior. In this regard, our results showed that subjects in the LL group had breakfast significantly later than individuals in any other group (*p* < 0.001) (Table 1), which was reflected as an increasing trend towards later breakfast timing as wakeup timing was delayed (*p* < 0.001). Noteworthy, the increments in the TDM were significantly associated with late wakeup timing (*p* = 0.011). Subjects in the EE group were those who, on average, had the shortest TDM (*p* < 0.001).

Regarding the association between sleep timing behavior and anthropometric markers of adiposity (Table 1), a significant trend towards lower BMI (*p* = 0.002), waist (*p* = 0.006), and hip circumference (*p* = 0.033) was found as the wakeup timing was delayed. Significant differences in BMI were found between extreme sleep timing behavior groups (EE versus LL). Subsequent analyses revealed that EE behavior was significantly associated with increased values of BMI and waist circumference (2.3 kg/m^2^ and 5.2 cm, respectively) when compared to the LL group (*p* < 0.05). These associations were independent of age, diet quality, total energy intake, and physical activity level.

Dietary intake and its association with sleep timing behavior is shown in Table 1. Of note, a significant trend towards a better diet quality was found as wakeup timing was delayed (*p* < 0.001). Interestingly, total energy intake was similar among groups, while a significant trend towards higher energy intake at dinner was found as the wakeup timing was delayed (*p* = 0.020). No differences were found between physical activity and sleep timing behavior.

**Figure 1:**
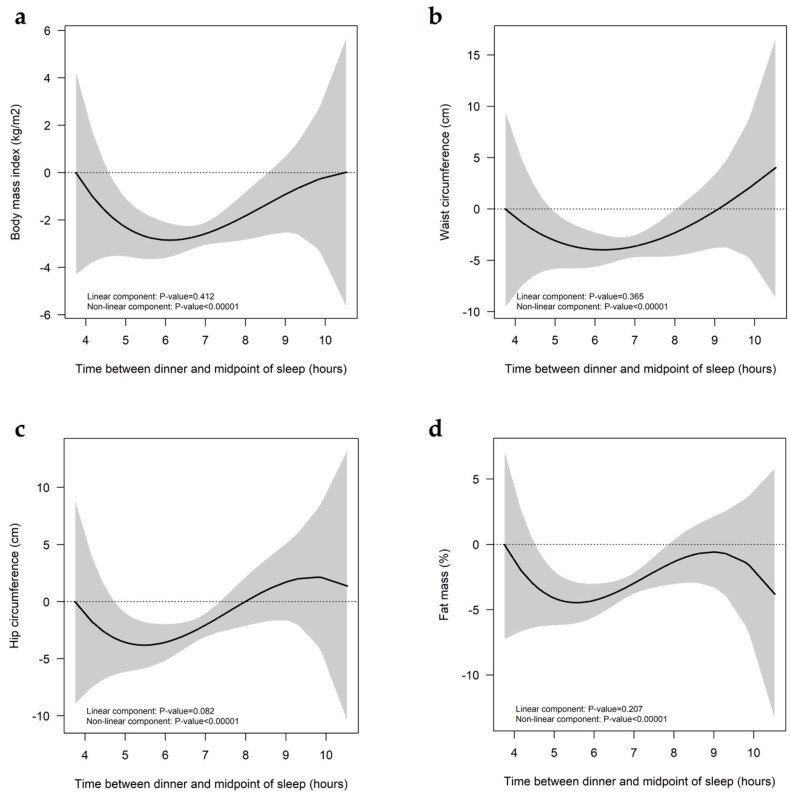
Restricted cubic splines representing the associations of the TDM with (**a**) body mass index, (**b**) waist circumference, (**c**) hip circumference, and (**d**) fat mass percentage. TDM, time elapsed between dinner and the midpoint of sleep. The models were adjusted for age, diet quality, total energy intake, and physical activity level. The gray band indicates the confidence levels for the regression line.

As summarized in Table 1, our results show that individuals in the EE group presented, on average, the shortest TDM, and had the highest BMI. Accordingly, the shorter the TDM, the higher the BMI. Hence, using the continuous values of these variables, we studied the association between TDM and adiposity measures. Results from multivariate regression analyses (adjusted for age, diet quality, total energy intake, and physical activity level) showed that associations of the 1 h increments in TDM with BMI (*p* = 0.079), waist circumference (*p* = 0.144), hip circumference (*p* = 0.081), and fat mass (*p* = 0.125) were not significant. Therefore, we studied the shape of these associations using restricted cubic spline models. As shown in Figure 1, a dose-response association was found between TDM and adiposity. Interestingly, a TDM of ~6 h was significantly associated with the minimal values of BMI, waist, hip circumference, and fat mass percentage. In particular, a TDM of 6 h could be related to approximately −3 kg/m^2^ of BMI, −4cm of waist and hip circumference, and −4.5% of fat mass.

## 4. Discussion

As far as we are aware, this is the first cross-sectional study to show an association between the timing of food intake relative to sleep/wake timing (expressed as TDM) and adiposity parameters in young women. To do so, we had two approaches. First, we compared differences between anthropometric parameters, eating patterns (dietary intake and meal timing), and physical activity according to sleep timing behavior categories. Then, we studied the shape of the association between the TDM and anthropometric markers of adiposity using restricted cubic splines. Our results revealed that individuals in the EE group had the shortest TDM, while they showed significantly higher values of BMI and waist circumference (2.2 kg/m^2^ and 5.2 cm, respectively), when compared to the individuals in the LL group. In addition, we observed a dose-response association between the TDM and adiposity, which showed that minimal adiposity values are observed when the subjects have dinner 6 h before their midpoint of sleep.

It is of interest that the LL group was the one with the lowest BMI despite having the highest percentage of energy intake at dinner. This fits with two other studies that have associated lower BMI values with later internal timing among young healthy adults [28,29]. Interestingly, Knutson et al. [28] pointed out that young adults were more likely to have a later internal timing and therefore, sleeping at a later clock time may align more closely with their circadian rhythms. Similarly, in our study, young women who had a LL behavior were more evening-oriented (shown by the midpoint of sleep). Thus, it is plausible that the alignment between internal circadian time and external clocks may mediate this relation, especially since their behavior would fit with their internal circadian rhythm.

Among the most relevant findings of our study is the fact that TDM was associated with adiposity markers. Noteworthy, on average, TDM was significantly shorter in individuals belonging to the EE group, which in turn showed the highest BMI. These results are in line with the observations of McHill et al. [3], who showed that eating closer to melatonin onset was associated with higher body fat percentage. To add, Xiao et al. [11] noted that among evening-oriented individuals, a higher percentage of energy intake 2 h before bedtime was associated with ~80% increase in the odds of being overweight or obese. Hence, with our results, we complement these observations, showing that the alignment between dinner time and the midpoint of sleep (given by the TDM) are relevant in maintaining a healthy weight and body composition.

As a first approach, we hypothesized that the concurrence between elevated glucose levels (due to postprandial state) and melatonin onset may occur through the association observed between EE behavior and adiposity, especially since melatonin levels begin to rise 2–3 h before habitual bedtime [30]. It is important to highlight that midpoint sleep is highly correlated with DLMO [15]. Recently, Lopez-Minguez et al. [14] pointed out that having dinner no later than 2 to 4 h before habitual bedtime would allow recovery of postprandial glycaemia to fasting values prior to the rise of endogenous melatonin levels. It is important to note that, when melatonin is bound to the Mel1b receptor in pancreatic-islet beta cells, it inhibits glucose-stimulated insulin secretion [14]. Therefore, having dinner near the midpoint of sleep may extend the postprandial glucose spike during the night. Although more evidence needs to be warranted, our results showed that a TDM of 6 h was associated with the lowest values of adiposity.

Additionally, some authors suggest that the thermic effect of food (the energy expended in response to a meal) and the respiratory quotient (which reflects macronutrient utilization) are lowest during a biological night [3,31,32]. Hence, another possible consequence of eating near DLMO is a decrease in the thermic effect of food, which in the long run could contribute to a positive energy balance and weight gain [3]. McHill et al. [3] observed that the timing of the caloric midpoint (defined by the average time by which 50% of daily calories are consumed) relative to DLMO was significantly associated with the percentage of body fat in young adults [3]. Accordingly, individuals with caloric midpoint closer to melatonin onset were those with the highest fat percentage. Together, these observations highlight the relevance of studying the timing of food intake relative to internal timing, rather than the clock hour. For this reason, we suggest that the TDM could be a practical marker of circadian misalignment. It is important to note that while dinner time was not modified by sleeping schedules, breakfast time changed.

Our data also reflect the pertinence of assessing whole sleep/wake patterns when evaluating the health status of the individuals. This includes not only duration or bedtime schedules, but also the alignment between sleep/wake cycles with social schedules, including weekend days. Interestingly, we observed a trend towards higher BMI, and waist and hip circumferences as wakeup timing was advanced. Recently, Wilms et al. [33] showed that sleep loss during the second half of the night decreased morning glucagon and cortisol levels. Although more evidence need to be warranted, the authors suggested that the timing of sleep restriction can potentiate its deleterious effects [33]. In addition, early wakefulness is related to impaired insulin sensitivity due to raised melatonin levels during the early-morning period [34]. In this regard, Eckel et al. [34] suggested that morning circadian misalignment may be a mechanism by which short sleep duration contributes to metabolic dysregulation and other health problems.

Regarding diet quality and its association with sleep timing behavior, our results show that individuals in both of the early-rise behavior categories (EE and LE) had the shortest sleep duration and the lowest diet quality. This fits with results from the Hispanic Community Health Study, in which short sleep duration was linked to poor dietary quality in adults [35]. Dashti et al. [8] reviewed this topic and pointed out that short sleep duration (<6 h) was associated with low diet quality. Together with insufficient sleep, other conditions that occur in parallel, such as increased hunger and food cravings for high-fat and/or sweet foods, as well as increased susceptibility to food rewards, may be potential mechanisms underlying these associations [8,36,37]. Although people do not always give into cravings, it is important to note that they have been associated with an excess of energy intake and obesity [36].

Taking into account the aforementioned, we consider that our findings raise interesting questions and novel opportunities for obesity prevention among college students or even teenagers, since their behavior is more evening-oriented [38]. In this regard, we showed that matching individual circadian preference (chronotype) with sleep timing behavior, as well as aligning meal timing with sleep/wake cycles, in particular dining ~6 h before the midpoint of sleep, could be related to lower adiposity. The latter could complement the recommendations of the World Health Organization for obesity prevention, which include limiting energy intake from fats and sugars, increasing the consumption of fruits and vegetables, as well as legumes, whole grains and nuts, and engaging in regular physical activity [39].

This study has several limitations, starting with its cross-sectional nature, which prevented us from finding causation. Furthermore, sleep habits (bed and wakeup timing) were assessed using sleep diaries and food intake with food logs, both of which are prone to be underreported. We also acknowledge that our female population is not representative of the entire population. Therefore, future studies should study the associations between the TDM and adiposity in men. In spite of this, our study has several strengths, including the fact that we collected detailed information on sleep and meal timing, as well as dietary intake on weekdays and weekends, which allowed us to capture habitual dietary and sleep patterns. Moreover, our study also assessed sleep and dietary quality.

## 5. Conclusions

In conclusion, our results show that both TDM and sleep timing behavior were associated with adiposity in young women. Individuals in the EE group showed a significant increase in BMI and waist circumference, when compared to the LL group. The latter could be explained by the misalignment between dinner timing and their internal timing (given by the TDM). This consideration could be of importance especially in young adults, or even teenagers, since their behavior is more evening-oriented. We showed that a 6-h TDM was significantly associated with lower adiposity. Hence, the TDM could constitute a practical approach to personalize the timing of food intake based on individual sleep schedules, which could be a useful strategy to align metabolism with circadian physiology. This, in the long run, could have a beneficial impact on BMI and adiposity, which is especially important for youngsters who are susceptible to a misalignment in sleep/wake timing and meal timing, and thus, need to establish healthy future habits.

## Figures and Tables

**Table 1:** Sleep- and circadian-related characteristics, anthropometric variables and dietary intake of the studied population grouped by sleep timing behavior.

	EE	LE	EL	LL	*p*-Value ^a^	*p*-Trend ^b^
n	34	33	33	33		
**Sleep Parameters**						
Wakeup time, hh:mm	06:32 (00:56) ^bc^	07:00 (00:52) ^de^	07:49 (00:33) ^bf^	08:39 (00:51) ^cef^	**<0.001**	**<0.001**
Bedtime, hh:mm	23:00 (00:37) ^ac^	00:30 (00:31) ^ade^	23:18 (00:31) ^df^	01:12 (00:45) ^cef^	**<0.001**	**<0.001**
Midpoint of sleep, hh:mm	02:49 (00:25) ^abc^	03:44 (00:27) ^ae^	03:52 (00:19) ^bf^	04:56 (00:30) ^cef^	**<0.001**	**<0.001**
Sleep duration, h	6.1 (0.9) ^bc^	5.7 (1.1) ^de^	7.2 (0.7) ^bd^	6.8 (0.9) ^ce^	**<0.001**	**<0.001**
Sleep quality	6.6 (2.5)	6.4 (2.8)	6.1 (2.9)	5.8 (3.0)	0.068	0.229
Social jet lag, h	1.1 (0.8)	1.1 (0.9)	1.1 (1.0)	1.3 (0.8)	0.350	0.372
**Meal Timing**						
Breakfast, hh:mm	08:34 (01:13) ^c^	08:23 (01:08) ^e^	08:58 (00:57) ^f^	9:46 (00:54) ^cef^	**<0.001**	**<0.001**
Lunch, hh:mm	15:30 (00:57)	15:36 (01:01)	15:18 (00:58)	15:06 (00:52)	0.159	0.060
Dinner, hh:mm	21:06 (00:49)	21:18 (00:58)	20:54 (00:51)	21:18 (00:53)	0.286	0.286
TDM, h	5.8 (0.9) ^abc^	6.6 (1.2) ^ae^	6.9 (0.9) ^bf^	7.6 (1.0) ^cef^	**<0.001**	**0.011**
**Anthropometric Parameters**						
BMI, kg/m^2^	25.4 (4.0) ^a^	23.8 (4.5)	23.0 (3.0)	22.5 (3.8) ^a^	**0.021**	**0.002**
Fat mass, %	32.2 (7.4)	31.5 (7.8)	30.5 (5.3)	29.5 (6.4)	0.387	0.082
Waist, cm	78.6 (8.8)	76.2 (9.7)	74.9 (8.4)	72.8 (7.4)	0.057	**0.006**
Hip, cm	99.5 (7.7)	97.3 (10.7)	96.3 (6.8)	95.2 (7.3)	0.194	**0.033**
**Dietary Intake**						
Diet quality	57.9 (6.8) ^ab^	60.7 (8.1) ^c^	64.0 (9.8) ^b^	67.3 (9.4) ^ac^	**<0.001**	**<0.001**
Total energy intake, kcal/day	1517 (404)	1596 (425)	1555 (412)	1676 (420)	0.452	0.179
Breakfast,% of kcal	24.8 (10.4)	26.9 (10.4)	26.5 (6.9)	22.8 (8.3)	0.258	0.381
Lunch, % of kcal	31.3 (7.5)	29.5 (10.2)	33.7 (10.5)	30.9 (9.6)	0.364	0.722
Dinner, % of kcal	18.0 (10.4)	18.6 (9.8)	20.7 (9.1)	23.5 (11.3)	0.123	**0.020**
Physical Activity, METs	1050 [1006; 2654]	1036 [462; 2026]	1040 [546; 2038]	1029 [447; 1893]	0.602	0.457

EE, early-bed/early-rise; LE, late-bed/early-rise; EL, early-bed/late-rise; LL, late-bed/late-rise; hh:mm, hours:minutes; TDM, Time elapsed between dinner and the midpoint of sleep. Values are mean (SD) and median [interquartile range] for non-normally distributed data. ^a^Statistical tests (ANOVA for normally distributed data or Kruskall-Wallis test for non-normally distributed data) were used to compare sleep parameters, meal timing, TDM, anthropometric and lifestyle parameters between sleep timing categories, followed by Bonferroni post-hoc comparisons between categories. Values with the same superscript in the same row are significantly different (EE vs. LE = a, EE vs. EL = b, EE vs. LL = c, LE vs. EL = d, LE vs. LL = e, EL vs. LL = f). ^b^ Pearson’s tests were used to calculate P-trend values. Significant *p*-values < 0.05 are shown in bold.

## Supplementary Materials

The following are available online at https://www.mdpi.com/2072-6643/12/2/410/s1, Table S1: General characteristics of the population studied.
