# Supplementary material for: The Elapsed Time between Dinner and the Midpoint of Sleep Is Associated with Adiposity in Young Women
Source: Nutrients. 2020 Feb 5;12(2):410. doi: 10.3390/nu12020410 (PMC7071164; doi:10.3390/nu12020410)
Supplement: Supplementary file 1 [file nutrients-12-00410-s001.pdf]

Supplementary Materials:

Table S1. General characteristics of the population studied.

	Mean (SD
n	133
Age (years)	19.9 (1.9)
**Anthropometric parameters**	
BMI (kg/m^2)	23.7 (4.0)
Fat mass (%)	30.9 (6.8)
Waist circumference (cm)	75.5 (8.7)
Hip circumference (cm)	97.5 (8.7)
**Dietary intake**	
Diet quality	62.4 (9.2)
Total energy intake (kcal/day)	1585 (413)
Breakfast (% of kcal)	25.1 (9.1)
Lunch (% of kcal)	31.5 (9.2)
Dinner (% of kcal)	20.2 (10.2)
**Physical activity (METs)**	531 [1036; 2109]
**Sleep parameters**	
Wakeup time (hh:mm)	07:29 (01:08)
Bedtime (hh:mm)	00:06 (01:36)
Midpoint of sleep (hh:mm)	03:49 (00:53)
Sleep duration (h)	6.4 (1.2)
Pittsburg sleep quality Index	6.3 (3.1)
**Meal timing**	
Breakfast (hh:mm)	08:55 (01:14)
Lunch (hh:mm)	15:19 (01:04)
Dinner (hh:mm)	21:09 (00:59)

**TDM (hours)**	6.7 (1.3)

METs: Metabolic Equivalent of Task; TDM: Time elapsed between dinner and the midpoint of sleep. Values are mean (SD) for normally distributed data and median [interquartile range] for non-normally distributed data
